# Cultural Intelligence Deployed in One’s Own vs. in a Different Culture: The Same or Different?

**DOI:** 10.3390/jintelligence11110212

**Published:** 2023-11-07

**Authors:** Robert J. Sternberg, Caleb Co, Ilaria Siriner, Arezoo Soleimani-Dashtaki, Chak Haang Wong

**Affiliations:** 1 Department of Psychology, Cornell University, Ithaca, NY 14853, USA; ccc283@cornell.edu (C.C.); asc329@cornell.edu (A.S.-D.); 2 Department of Psychology, Syracuse University, Syracuse, NY 13244, USA; isiriner@syr.edu; 3 Teachers College, Columbia University, New York, NY 10027, USA

**Keywords:** cultural intelligence, intelligence, maximum-performance test, typical-performance test

## Abstract

Cultural intelligence is one’s ability to adapt when confronted with problems arising in interactions with people or artifacts of cultures other than one’s own. In this study, we explored two maximum-performance tests of cultural intelligence. One, used in previous research, measured cultural intelligence in the context of an individual conducting a business trip in another culture. The second, new to this research, measured cultural intelligence in the context of meeting someone from another culture while one is in the context of one’s own culture. So, the difference between the two tests was whether one was in one’s own culture or another and whether the individual who most had to adapt was oneself or someone else. We found that cultural intelligence in the two contexts was essentially the same construct. Cultural intelligence as measured by a typical-performance test is a different construct from cultural intelligence as measured by a maximum-performance test. In this research, general intelligence showed some limited correlation with cultural intelligence as measured by a maximum-performance, but not a typical-performance test. Cultural intelligence as an ability and as a disposition are not the same but rather complement each other.

## 1. Introduction

Many of us who have traveled cross-culturally have experienced culture shock as a result of cultural differences. Here are some of the questions we, the coauthors, have asked ourselves: “Why are people in this country always seemingly so grumpy?”, “Why did no one laugh at my great joke, which people always laugh at in my culture?”, “Why do they have to drive on that side of the road—I almost got run over?!”, “Why are they all staring at me?”.

In today’s global world, being able to relate positively and effectively to people from other cultures is more important than ever before. Adapting to another culture virtually always requires a serious intellectual and, sometimes, emotional effort. This experience is different from adapting to new people within one’s own culture because, although meeting new people within one’s own culture can be a challenge, one likely shares many cultural assumptions and cultural tacit knowledge that is not shared with someone from a different culture. Moreover, a spoken line, a gesture, or a facial expression that means a certain thing in one’s own culture may mean something else in another culture. So, one may think one is communicating one thing when, in fact, one is communicating something alien to the intended message.

Cultural intelligence is “one’s ability to adapt when confronted with problems arising in interactions with people or artifacts of cultures other than one’s own” (Sternberg et al. 2021). People who are culturally intelligent are able to function effectively in culturally diverse environments. Cultural intelligence is different from general intelligence, as it pertains specifically to intercultural interactions—ones in which one’s tacit knowledge and presuppositions about human behavior may be violated, perhaps with regularity. Cultural intelligence is important in a world in which intercultural experiences and negotiations have become part of many people’s everyday existence. The differences between cultures need not be international, as shown by the incomprehension experienced in many instances between people living in “red” (conservative) and “blue” (liberal) states of the U.S., or some of the conflicts between groups such as Hindus and Muslims in India.

Bennett (1986, 2017) has proposed a developmental model of intercultural sensitivity that is certainly relevant to the notion that people can develop cultural intelligence, or at least, attitudinal aspects of it. According to Bennett, intercultural sensitivity can develop through six stages: (a) denial; (b) defense; (c) minimization; (d) acceptance; (e) adaptation’ and (f) integration. In the last stage, one has fully integrated cultural differences into one’s perceptions so that the sense of strangeness has been replaced by a sense of welcome of the differences. An Intercultural Development Inventory (IDI) measures the stages of the development of intercultural sensitivity (Hammer 2012; Hammer and Bennett 1998; Hammer et al. 2003). Stemler et al. (2014) have created a situational judgment test (SDT) that measures levels in the Bennett model. Corbitt (1998) has offered a Global Awareness Profile (GAP) that consists of 120 multiple-choice items.

Two major approaches, represented by the tests cited above, have been used to measure cultural intelligence—typical performance and maximum performance. It should be said at the outset that the approaches are not mutually exclusive or even incompatible. The typical-performance approach asks participants to characterize their everyday thoughts, feelings, and behaviors (Ang et al. 2006, 2007, 2015, 2020; Van Dyne et al. 2008). The maximum-performance approach presents situations requiring the exercises of cultural intelligence and has participants solve intercultural problems (Sternberg et al. 2021, 2022; Chen 2020; Schwarzenthal et al. 2019). The typical-performance approach measures cultural intelligence as a disposition; the maximum-performance approach measures it as an ability. Both dispositions and abilities are presumably necessary in order to cope with novel intercultural situations. One must want to deal thoughtfully with situations (dispositions) but also have the skills required to deal with them effectively (abilities).

Both typical-performance and maximum-performance measures of cultural intelligence show good reliability and validity. Past maximum-performance tests measuring cultural intelligence have been correlated to other variables. For example, Schwarzenthal et al. (2019) created a measure using several situational judgment tests (SJTs) asking to provide a response to intercultural scenarios. Although the test contained a limited number of SJT’s, the researchers found a positive correlation to a self-reported cultural intelligence test adapted by Van Dyne et al. (2012). Chen (2020) developed two tests using both SME (small and medium sized enterprise)-based SJTs and model-based SJTs. SME-based SJTs were constructed in the form of an interview of cultural scenarios, while the model-based SJTs were structured in an open-ended questionnaire. Both of these tests displayed convergent validity to a typical Cultural Intelligence Scale (CQS) developed by Van Dyne et al. (2008), but the SME-based SJTs performed higher than the model-based SJT’s in predicting multicultural performance in teams.

Sternberg et al. (2021, 2022) found that although the typical- and maximum-performance approaches to measurement both show sensible patterns of correlations with other variables, the tests deriving from them do not tend to correlate significantly with each other. In general, perhaps somewhat predictably, typical-performance tests of cultural intelligence tend to correlate with other relevant variables that are measured through typical-performance tests, and maximum-performance tests of cultural intelligence tend to correlate with other relevant variables that are measured through maximum-performance tests. For example, the typical-performance approach uses measures such as a measure of openness to experience, whereas the maximum-performance tests of cultural intelligence tend to correlate with measures of fluid intelligence and with other maximum-performance measures of cultural adaptability. Maximum-performance items that measured cultural intelligence in business and leisure settings were very highly correlated, suggesting that, for the most part, people who are adept at solving intercultural problems in business settings also are adept at solving them in leisure settings, and vice versa. The past results suggested that cultural intelligence draws on general intelligence but is by no means the same thing as general intelligence as conceived of by Carroll (1993), Deary (2020), McGrew (2009), and others.

The maximum-performance test items used by Sternberg et al. (2021, 2022) asked questions about an individual visiting another culture, either for business or leisure reasons, and how they would respond to intercultural problems in the context of such a visit. However, many of the inter-cultural problems people in a given dominant culture confront are not in the context of visiting another culture but rather in the context of interacting with individuals from another culture in the people’s own, dominant culture. In these instances, they are the ones whose culture dominates in the present context, and they are trying to work with someone for whom their dominant culture is strange. What is the relationship between cultural intelligence in the context of one’s own dominant culture versus in the context of visiting another place where some other culture is dominant?

The answer is not immediately obvious because of the role reversal this situation involves. In one situation, one has to adapt to a set of customs that may seem culturally alien; whereas, in the other situation, one has to adapt to another person who is dealing with a potentially culturally alien situation. In both cases, one has an intercultural challenge, but in essentially opposite situations. The two aspects of cultural intelligence might be quite similar because they both involve interacting with people of another culture; but they might be quite different because in one situation, the burden is on oneself to adapt to a novel cultural situation, whereas in the other situation, the burden is on another to adapt to the novel situation. In the latter situation, one is adapting to the other’s less than complete success in understanding one’s own culture.

In a sense, the difference may be roughly analogous to the hometown advantage in athletic events. Teams playing in their hometown stadium have the advantage of being familiar with the setting, knowing many aspects of the context in which they are operating, having a supportive cast or audience around them, feeling more comfortable, and, usually, not having to deal with jet lag or other stresses arising from being in a new setting. In each case, one is dealing with others, but the challenges are different when one is doing so on the other’s home turf rather than one’s own. A further example would be the difference between negotiating with foreign ambassadors either in one’s home country or in the negotiating partner’s home country. The former is easier than the latter, as the rules that apply within one’s own country prevail. In general, negotiating on home turf is easier (Harvard Program on Negotiation 2023).

The present study was intended to address, and hopefully answer, the question of the relationship between the two kinds of cultural intelligence, i.e., that applied outside one’s own culture and inside one’s own culture. Our expectation was that the skills would be closely related, but to our knowledge, no such study has been conducted before, so we had no data on which to base the prediction. Our expectation of a high correlation was based on the theory of adaptive intelligence (Sternberg 2019), according to which both situations would involve adapting one’s behavior in order to serve the common good of the participants in the interaction. We tested participants with both our previous, old scale and a new modified scale that presented cultural intelligence problems in the context of one’s own culture, with members of another culture.

## 2. Method

### Participants

A total of 148 undergraduate and graduate students studying at a highly selective university near the East Coast of the U.S.A. participated in an online survey for data collection. The majority of these participants were female (112), while the rest were male (36). The average age of the participants was 20.15, with a standard deviation of 1.61.

## 3. Materials

There was a total of 9 assessments in the online survey. The assessments were created with Qualtrics and administered through Sona Systems. These assessments consisted of two psychometric tests: Letter Sets and Figure Classifications; two Maximum-Performance Cultural Intelligence Tests created by the researchers, in which one simulated scenarios in a different culture (CIB-Original) while the other simulated scenarios of a colleague’s experiences in one’s own culture (CIB-Modified); a Views on Culture Questionnaire (VC); a typical Cultural Intelligence Scale (CQS); a Diagnosing Your Cultural Intelligence Test from the *Harvard Business Review* (HCT); a Test of Personal Intelligence (TOPI); and a demographic questionnaire.

**Psychometric Assessments.** The two psychometric assessments from this study were as follows: (1) Letter Sets, in which participants selected one set of letters that did not match the patterns/properties of four other Letter Sets; and (2) Figure Classification, in which participants were shown different groups of figures separated by different properties and then assigned additional figures into one of those groups based on those properties.

Both of these assessments were adapted from The Kit of Factor-Referenced Cognitive Tests (Ekstrom et al. 1976), which measures intelligence and related abilities. The items were scored based on the number of correct answers, with one point given for each correct response. The participants were given seven and eight minutes, respectively, to complete the psychometric assessments.

**Maximum-Performance Cultural Intelligence Tests (CI).** Two versions of the Cultural Intelligence Tests were developed (which are available immediately upon request from the first author of this article). The first test (the Cultural Intelligence Business Test Original, or CIB-Original) simulated scenarios in which the participant was on a business trip representing his or her home country and traveled to a country with a different culture. The second test (the Cultural Intelligence Business Test Modified, or CIB-Modified) simulated scenarios in which the participant was the boss of a new employee who had just arrived from a different country with a different culture from the participant. In both versions of the test, which we refer to as the Sternberg Cultural Intelligence Test, the participants were first faced with several conflicts representing different cultural contexts. The test is not standardized or normed. The participants were then asked what they would do if they were in that scenario. One example from the CIB-Original is shown below:

*“After taking a long overnight flight, two short connecting flights, and a 5-hour bus ride, you have finally arrived. You feel absolutely exhausted and want to head straight for the hotel to rest and get refreshed. However, the person who comes to pick you up from the bus station seems to be in a hurry and wants you to be in a meeting as soon as possible. You do not feel like you are ready for a meeting yet. If there are any decisions to be made, you would not even trust yourself. What would you do?”*

One example from the CIB-Modified is shown below:

*“You have assigned your new employee a project to work on that is due in one week. You emphasize that if they have any questions, they should not hesitate to reach out and ask. After one week, you receive their report. You have found significant communication problems, not only in their writing but also in their understanding of the tasks. You are aware that their first language is not English; however, you still feel frustrated that they never came to you with any questions or for any clarifications. What would you do?”*

There were twelve items each for both the CIB-Original and CIB-Modified. Each item was graded by four different graders, with the final score representing the average of the four graders. Each of the graders responded independently of each of the others. The scoring was based on a five-point scale, with “1” indicating a poorly answered item and “5” indicating a very well-answered item. (No “5” ended up being given in the grading of either the CIB-Original or the CIB-Modified.) Participants were graded for both the quantity and quality of their responses. However, the quantity of responses could not make up for the lack of quality. For example, suppose a participant wrote for the CIB-Modified, “I would tell them to suck it up and learn to communicate with Google, read books, and use ChatGPT because their culture is deficient in that area of learning.” That participant would score a “1” despite giving multiple suggestions because the quality of the suggestions represented an ethnocentric point of view instead of relevant points that utilize cultural intelligence. The grading rubric for both tests is shown below.
**Rating****# of Suggestions****Elaborates Suggestions****Gives Objective Examples****Sample**0No answer/irrelevant answer11
-I would go to a hospital21–2Some limited explanations-I would go use hand gestures to indicate my illness and ask for a map to find a hospital32 or moreSome limited explanations-I would first do…, then…; if something went wrong, I would…43 or moreElaborated explanationsYesI would use nonverbal body language to show that my stomach is in pain. If there was a pharmacy nearby, I would point to that to a local and then use nonverbal body language to see if a local could help me find the hospital. If that did not work, I would pretend to be listening to someone’s heartbeat with a stethoscope and see if someone could help me find a hospital after that.53 or more plausible (novel and unique)Specific and detailed explanationsYes(Something valid that is not mentioned by other people or that is mentioned infrequently.)

Raters were extensively trained in the use of the rubric. The inter-rater reliability for the four graders was 0.98 for CIB-Original, 0.99 for CIB-Modified, and 0.99 for CIB-Original and CIB-Modified together. The results are comparable to Sternberg et al. (2022), in which the inter-rater reliability was 0.98 among the three graders; as well as to Sternberg et al. (2021), in which the inter-rater reliability was 0.99+ among the two graders.

**Views on Culture (VC).** The Views on Culture questionnaire consisted of three items that gauged the participant’s interests and personal opinions of different aspects of culture. Each item is listed below:

*Item 1*: “Some people believe it is worthwhile to learn to speak at least one foreign language fluently. Other people believe it is not worthwhile.

*a.*  *What do you believe?*
*b.*  *Give the reasons why you believe what you believe.”*

*Item 2*: “Some people believe it is worthwhile spending a significant amount of time (at least six months) living in a foreign country. Other people believe it is not worthwhile.

*a.*  *Have you ever lived in a foreign country for at least six months?*
*b.*  *What do you believe?*
*c.*  *Give the reasons why you believe what you believe.”*

*Item 3*: “You meet someone from a foreign country who, in a conversation, expresses beliefs with which you strongly disagree. You are surprised that they could believe and express such a thing.

*a.*  *What would you say or do?*
*b.*  *Why would you say or do that?”*

The grading for the Views on Culture items was as follows: Item 1a was not given a score; Item 1b was graded on a three-point scale assessed by the number of reasons given and the quality of the reasons; Item 2a was graded with “yes” as one point and “no” as zero points; Item 2b was not given a score; Item 2c was scored on a three-point scale for the number and quality of the responses; Items 3a and 3b were scored together as a whole answer. They were graded on a three-point scale for the number and quality of responses. The rubric for the items scored on a three-point scale was as follows.
**Rating****Description**0No answer/perverse answer (irrelevant/mean)1Weak response (ex. “I don’t understand why you would say that.”)2Good answer (in quantity or quality of responses, e.g., “I would say that is an inappropriate thing to say; I would bring other friends over and talk about it, and I would distance myself from that person.”)3Very good answer (in quantity and quality of responses, e.g., “I would talk to that person and maybe other friends nearby about why they believe that to get a better understanding, and then I would explain what I believe and we could talk about why we disagree so I could potentially change their mind, or they change mine.”)

These items were also graded by four separate graders, with the final score the average of the four grades. The inter-rater reliabilities, computed as intraclass correlations coefficients, for the three Views on Culture Items were 0.85, 0.83, and 0.90, respectively.

**Typical-Performance Cultural Intelligence Scale (CQS).** The Cultural Intelligence Scale is an already validated measure of cultural intelligence created by Van Dyne et al. (2008). This scale assesses participants’ cultural knowledge, values, and adjustment to different norms. This scale has four dimensions namely (1) Metacognitive, (2) Cognitive, (3) Motivational, and (4) Behavioral. An example of an item on this test is “I am conscious of the cultural knowledge I apply to cross-cultural interactions.”

This statement is a part of twenty statements that are rated on a 7-point Likert scale (1 = strongly disagree; 2 = disagree; 3 = more or less disagree; 4 = undecided; 5 = more or less agree; 6 = agree; 7 = strongly agree), with higher self-reported scores demonstrating a higher level of cultural intelligence. Based on the data collected in this study, the CQS showed a reliability of α = 0.93 across 20 items.

**Diagnosing Your Cultural Intelligence Test (HCT).** The *Harvard Business Review* published a test created by Professors Earley and Mosakowski (2004) called the “Diagnosing Your Cultural Intelligence” Test, which is a 12-item questionnaire designed to assess the participants’ cultural efficacy. For example, one item on this test queries if the participant often asked themselves what they wanted to achieve or to obtain from a conversation whenever they were interacting with a person from another culture. Each item is self-reported by the participant on a five-point scale (1 = strongly disagree, 2 = disagree, 3 = neutral, 4 = agree, 5 = strongly agree), with higher numbers correlating to a higher cultural intelligence score. This study’s data showed that the HCT had a reliability of α = 0.86 across 12 items.

**Test of Personal Intelligence Mini–12 (TOPI).** The Test of Personal Intelligence used in this study is a condensed version of the full TOPI questionnaire made by Mayer et al. (2018). The TOPI used in this study contains twelve items that assess the maximum-performance problem-solving skills of students through a list of questions in a multiple-choice format. Based on the data collected in this study, this test had a reliability of α = 0.82 across 12 items.

**Demographic Questionnaire.** To conclude the study, the participant was asked to fill in several demographic questions, such as age, gender, class year, first language, SAT and ACT scores (if they took either or both of the tests), and GPA, as well as questions pertaining to experience with other cultures, such as the amount of contact with other cultures, number of different countries visited, and cross-cultural experiences in years.

## 4. Design

The design of this study was correlational. The dependent variables were the cultural intelligence tests (CIB-Original and CIB-Modified). The independent variables were the other ability tests. The design was completely within-subjects.

## 5. Procedure

Students from a highly selective university near the East Coast of the U.S.A. were recruited through Sona Systems and presented the online survey through Qualtrics. Participants were asked to sign an informed-consent form before starting the study. After signing, the participants began the two psychometric assessments (Letter Sets and Figure Classification), with a seven-minute and eight-minute time limit, respectively. After the tests were completed, participants completed, in order, the Cultural Intelligence tests (CIB-Original and CIB-Modified), Views on Culture (VC), Cultural Intelligence Scale (CQS), Diagnosing Your Cultural Intelligence Test from *Harvard Business Review* (HCT), Test of Personal Intelligence (TOPI), and the demographic questionnaire, all of which did not have a time limit. After completing all the items, the participants were shown a debriefing form. The entire study lasted no longer than 1.5 h.

## 6. Results

### 6.1. Basic Statistics

Descriptive statistics for demographic questions (age, cross-cultural experience in years, and number of countries visited), psychometric assessments (Letter Sets, Figure Classification, and TOPI), standardized admissions tests (ACT and SAT with subtests reading and math), and college GPA are summarized in Table 1. Table 1 further provides basic statistics for the tools that were used to assess cultural intelligence: the business-subtest of the maximum-performance Sternberg Cultural Intelligence Test (Total CI)—including the two subtests, the *original* subtest from earlier research (Sternberg et al. 2021, 2022) for visiting another culture (CIB-Original), as well as a *modified* subtest for someone from another culture visiting one’s own culture (CIB-Modified). There also were three items that assessed Views-on-Culture (VC), the Diagnosing Your Cultural Intelligence Test (HCT) by Earley and Mosakowski (2004), and the typical-performance Cultural Intelligence Scale (CQS) by Van Dyne et al. (2008).

Note that the CIB-Original and the CIB-Modified both presented maximum-performance cultural-intelligence challenges. The difference was whether the encounter with one or more individuals from another culture occurred in the other’s country (CIB-Original) or in one’s own country (CIB-Modified). In each case, the described individual had to adjust to someone from another culture: what differed was the context in which the encounter took place.

On average, participants have visited 7.15 countries, with a standard deviation of 6.80, while the average time spent in a country other than their own was 7.15 years, with a standard deviation of 7.60.

For psychometric assessments, the mean score for Letter Sets was 10.01 (SD = 3.42), and for Figure Classification, it was 66.03 (SD = 20.93); lastly, participants averaged 9.60 on the test of personal intelligence (TOPI) with a standard deviation of 2.76.

Among the 148 participants, 65 provided ACT scores, ranging from 17 to 36 (M = 32.26; SD = 3.44), and 94 provided SAT scores for Reading (M = 721.76; SD = 59.77) and Math (M = 766.01; SD = 49.68). The conversion from SAT to ACT scores were reported by 126 participants, with scores ranging from 18 to 36 (M = 33.18; SD = 2.82). We received GPA scores from 129 participants, ranging from 3.0 to 4.0, with a mean of 3.65 (SD = 0.37).

Mean ACT and SAT scores in our population were higher than the average population of college students, with the national ACT average of 19.8 (https://www.act.org/content/dam/act/unsecured/documents/2022/2022-Average-ACT-Scores-by-State.pdf accessed on 13 August 2023) and the national SAT averages of the SAT Reading of 533 and the SAT Math of 527 (https://nces.ed.gov/programs/digest/d17/tables/dt17_226.40.asp accessed on 13 August 2023). Our sample also featured smaller standard deviations in SAT scores compared with the national standard deviations of 100 and 107. However, many participants did not take or report the standardized tests; these might be students who would have scored or did score lower and thus chose not to take or report standardized tests.

With regard to measures of cultural intelligence, participants’ scores on the business-subtest of the Sternberg Cultural Intelligence Test (Total CI) were obtained, with subtest scores for visiting another culture (CIB-Original) averaging 27.81 (SD = 8.20), and for someone from another culture visiting (CIB-Modified) averaging 27.72 (SD = 8.52). The total (summed) cultural intelligence score (Total CI) ranged from 23.00 to 117.75, with a mean of 55.53 (SD = 16.04). Participants’ Views-on-Culture (VC) were assessed using three items. The average scores for VC Item 1, VC Item 2, and VC Item 3 were 1.72 (SD = 0.59), 1.89 (SD = 0.57), and 1.88 (SD = 0.62), respectively.

The Diagnosing Your Cultural Intelligence Test (HCT) yielded scores ranging from 22.00 to 60.00, with a mean of 41.60 (SD = 7.07). The Cultural Intelligence Scale (CQS) scores ranged from 34.00 to 140.00, with an average of 90.32 (SD = 18.87). Regarding the CQS dimensions, participants’ scores on Dimension 1: Motivational (MOT); Dimension 2: Cognitive (COG); Dimension 3: Behavioral (BEH); and Dimension 4: Metacognitive Cognition (MOT), were as follows: 20.62 (SD = 4.08), 22.37 (SD = 7.37), 24.47 (SD = 5.66), and 22.85 (SD = 6.69), respectively.

### 6.2. Analyses of Variance

A multivariate analysis of variance (MANOVA) was conducted to investigate the impact of gender on the dependent variables. The results revealed that collectively, gender did not exert a statistically significant influence on this set of dependent variables (*F* (17, 12) = 1.530 (*p* = 0.25); Wilk’s Λ = 0.278).

A second MANOVA examined the influence of participants’ first language (English, Chinese/Mandarin, Korean, Bilingual, Spanish, Polish, Thai, Bengali) on multiple dependent variables. The MANOVA did not reveal a significant overall effect of first language on the combined dependent variables (Wilk’s Λ = 0.381; *F*(11, 17) = 1.100 (*p* > 0.05)).

The third MANOVA was conducted to investigate the impact of participants’ level of contact with people from other cultures on a range of dependent variables. The overall MANOVA test did not indicate a significant effect of the level of cultural contact on the combined dependent variables (Wilk’s Λ = 0.020, *p* > 0.05).

### 6.3. Internal Consistency Reliabilities

Table 2 presents the coefficient *α* values, indicating the internal consistency reliabilities of the tests. The cultural intelligence tests we developed, measuring maximum performance, demonstrated robust internal consistency, with coefficient *α* values of 0.96 for both the “own culture” and “foreign culture” subscales, as well as 0.98 for the total score. These reliabilities were high and either comparable to, or greater than, those of the other measures, such as the Cultural Intelligence Scale (CQS) at 0.93 and the Diagnosing Your Cultural Intelligence Test (HCT) at 0.86 with a comparable number of items. Notably, these internal-consistency reliabilities were higher than those reported in earlier iterations of the test; specifically, the revised Sternberg et al. (2022) version achieved a coefficient alpha of 0.97 for the total score, surpassing the initial version (Sternberg et al. 2021), with a coefficient alpha of 0.87. This improvement could potentially be attributed to the test’s revision and extension.

### 6.4. Intercorrelations

Pearson correlation coefficients were calculated to explore the relationships between various psychometric tests, typical performance measures of cultural intelligence, maximum performance measures of cultural intelligence, views on culture, and cross-cultural experience. Table 3 presents the correlation matrix. We anticipated finding significant correlations among the maximum performance measures and among the typical performance measures, but not necessarily between the two. In addition, we were further interested in whether cross-cultural interactions within a foreign culture (CIB-Original) correlated with the same or different variables from those within own’s culture (CIB-Modified).

### 6.5. Academic Performance Related Measures

The statistical results presented in this section demonstrate the relationships between various psychometric assessments and their associations with academic performance and cultural intelligence. Furthermore, significant positive correlations were found between ACT scores and cognitive tasks such as Letter Sets (*r* = 0.29, *p* < 0.05), Figure Classification (*r* = 0.38, *p* < 0.01), CQS Dimension 2 (*r* = 0.26, *p* < 0.05), and TOPI (*r* = 0.25, *p* < 0.05). Similarly, SAT Reading scores were positively correlated with GPA (*r* = 0.39, *p* < 0.01), as well as with CIB-Original (*r* = 0.23, *p* < 0.5), total CI (*r* = 0.23, *p* < 0.5), and Views on Culture item 3 (*r* = 0.21, *p* < 0.5). Additionally, SAT Math scores exhibited a positive correlation with GPA (*r* = 0.22, *p* < 0.05).

These results suggest a link between psychometric assessments and academic performance. Unlike the previous version of the Cultural Intelligence Test (Sternberg et al. 2021), this updated version revealed some significant correlations with SAT reading scores. There may have been a greater range of participant skills in the present sample, or the sample may have drawn on slightly different sets of skills in solving the cultural items. Another possible explanation for this disparity could be attributed to a different range of scores within the current sample.

### 6.6. Psychometric Tests

In the realm of psychometric assessments, beyond the aforementioned correlations, further observations were made. Specifically, Letter Sets displayed positive associations with Figure Classification (*r* = 0.50, *p* < 0.01), TOPI (*r* = 0.55, *p* < 0.01), maximum performance cultural intelligence test (*r* = 0.36, *p* < 0.01), CIB-Original (*r* = 0.34, *p* < 0.01), and CIB-Modified (*r* = 0.36, *p* < 0.01) subscales. Additionally, Letter Sets were correlated with all three views on culture items: VC_Item1 (*r* = 0.41, *p* < 0.01), VC_Item2(*r* = 0.23, *p* < 0.01), and VC_Item3 (*r* = 0.25, *p* < 0.01). Although no significant relationship emerged between Letter Sets and the typical performance measure CQS, contrary to our hypothesis, a positive correlation was observed with the HCT (*r* = 0.17, *p* < 0.05).

Furthermore, Figure Classification demonstrated significant correlations with CIB-Original (*r* = 0.22, *p* < 0.01), CIB-Modified (*r* = 0.23, *p* < 0.01), Total CI (*r* = 0.24, *p* < 0.01), the first (*r* = 0.31, *p* < 0.01), and the third (*r* =0.18, *p* < 0.05) views on culture items. This correlation was not present for the second view. Additionally, Figure Classification exhibited a positive correlation with the fourth CQS item (*r* = 0.22, *p* < 0.01), as well as correlations with the HCT (*r* = 0.26, *p* < 0.01) and TOPI (*r* = 0.44, *p* < 0.01).

TOPI also displayed a positive association with the number of countries visited (*r* = 0.25, *p* < 0.01).

The results indicate that Letter Sets appear to have a strong connection with maximum, but not the typical performance cultural-intelligence measure CQS. On the other hand, Figure Classification demonstrates significant associations with various cultural intelligence measures.

### 6.7. Maximum Performance Measures of Cultural Intelligence

For the maximum-performance cultural intelligence test, and in line with our hypothesis, CIB-Original exhibited positive and significant correlations with the three views on culture items: VC_Item1 (*r* = 0.47, *p* < 0.01), VC_Item2 (*r* = 0.35, *p* < 0.01), and VC_Item3 (*r* = 0.44, *p* < 0.01), as well as with TOPI (*r* = 0.38, *p* < 0.01). Similarly, CIB-Modified showed strong positive correlations with VC_Item1 (*r* = 0.54, *p* < 0.01), VC_Item2 (*r* = 0.51, *p* < 0.01), and VC_Item3 (*r* = 0.60, *p* < 0.01), the second item of CQS (*r* = −.19, *p* < 0.05), and TOPI (*r* = 0.47, *p* < 0.01).

The three Views on Culture (VC) items primarily exhibited correlations among themselves. Moreover, the second VC item showed a correlation with the fourth CQS item (*r* = 0.19, *p* < 0.05), and all three VC items correlated with TOPI (*r* = 0.39, *p* < 0.01) for VC_Item1, (*r* = 0.41, *p* < 0.01) for VC_Item2, and (*r* = 0.36, *p* < 0.01) for VC_Item3, respectively.

As with the last iteration of the Sternberg Cultural Intelligence Test, both CIB-Original and CIB-Modified are strongly correlated with cultural views (VC_Item1, VC_Item2, VC_Item3) and personal intelligence (TOPI). Additionally, the cultural views items (VC) correlated with each other and with TOPI.

### 6.8. Typical Performance Measures of Cultural Intelligence

Supporting our hypothesis, the HCT was strongly correlated with total CQS (*r* = 0.79, *p* < 0.01), CQS dimensions 1 (*r* = 0.63, *p* < 0.01), 2 (*r* = 0.50, *p* < 0.01), 3 (*r* = 0.72, *p* < 0.01), and 4 (*r* = 0.71, *p* < 0.01), as well as with the number of countries visited (*r* = 0.25, *p* < 0.01). The total score on the CQS demonstrated significant correlations with all its dimensions. Furthermore, correlations emerged between the CQS and the number of countries visited (*r* = 0.26, *p* < 0.01), as well as the number of years of cross-cultural experience (*r* = 0.25, *p* < 0.01). The first (motivational cultural intelligence) and fourth (behavioral cultural intelligence) dimensions of the CQS exhibited correlations with years of cross-cultural experience (*r* = 0.18, *p* < 0.05) and (*r* = 0.32, *p* < 0.01), as well as the number of countries visited (*r* = 0.22, *p* < 0.01) and (*r* = 0.22, *p* < 0.01), respectively. Lastly, the third dimension showed a correlation with the number of countries visited (*r* = 0.27, *p* < 0.01).

The results reveal strong correlations between the typical performance tests of cultural intelligence as well as their subscales. Real-world cross-cultural exposure, represented by the number of countries visited and years of experience, also positively correlates with CQS scores, suggesting that the more cultural experiences one has, the higher the score on the CQS.

### 6.9. Principal Component Analyses

This section presents the outcomes of the principal component analyses. Unless explicitly indicated, the results of the principal factor analyses matched those obtained through principal component analysis. To access the findings of the factor analysis, please write to the senior author by email and the results will be sent promptly.

Separate principal component analyses were conducted for different subsets of tests because listwise deletion of cases with missing data, if all the tests were used, would potentially result in deletion of large numbers of cases.

Displayed in Table 4 are the results of a principal component analysis of psychometric measures (Letter Sets, Figure Classification, TOPI), maximum performance (Total CI, VC items 1–3), and typical performance cultural intelligence tests (HCT, CQS). Total CI and VC maximum-performance measures constituted the first factor, psychometric tests formed the second, and typical-performance CQS and HCT comprised the third.

Table 5 provides the results of a principal component analysis of the TOPI, the maximum-performance measures CIB-Original, CIB-Modified, and all three Views on Culture Items, the academic variables SAT to ACT conversion and GPA, as well as the typical performance cultural intelligence measures HCT and CQS. Three components had Eigenvalues greater than one: the maximum performance cultural intelligence measures and TOPI made up the first component, the typical performance cultural intelligence measures the second, and the academic variables the third.

Table 6 presents a parallel analysis to that in Table 5, involving a principal component analysis but with the inclusion of the Total CI score in place of the two individual subtests. The other variables remain constant, encompassing the three views on culture items, CQS, HCT, as well as TOPI, SAT to ACT conversion, and GPA. Much like the findings in Table 5, the first component in Table 6 is formed by the Total CI score, the three views on culture items, and TOPI. The second component continues to represent the typical performance cultural intelligence measures, while the third component is characterized by the SAT to ACT conversion and GPA.

Table 7 presents the results of a principal component analysis using CIB-Original, CIB-Modified, and the three views on culture items as maximum performance cultural intelligence measures, the typical performance cultural intelligence measures CQS and HCT, the psychometric tests TOPI, Letter Sets, and Figure Classification, as well as the academic variables SAT to ACT conversion and GPA. The first component is made up of the maximum performance cultural intelligence measures, while the third is made up of the typical performance ones. The second factor includes the psychometric tests and the TOPI and the fourth includes the academic variables.

## 7. Discussion

Cultural intelligence is essential in today’s world, especially as people travel more across cultures and also as accommodating immigration—legal and illegal—becomes a greater challenge practically everywhere in the world. Our study showed cultural intelligence, at least as we measured it, to be pretty much the same whether it is employed within one’s own or in another culture.

People are visiting and moving in large numbers from one country to another. More than 40 million out of 330 million people living in the United States, for example, were born elsewhere. Roughly one-quarter of immigrants are illegal (Budiman 2020). In some parts of the United States and other countries, it is almost impossible to live one’s daily life without interacting with people from other cultures. Schools teach a great deal about subjects with which their students will have little or no contact outside school. They teach little about interacting with visitors to the country and with immigrants, legal or otherwise. In addition, in many jobs, good performance has come to depend on cultural skills.

Our study showed that cultural intelligence can be measured reliably and, at least according to the measures of convergent and discriminant validity we used, validly. Cultural intelligence is a different entity when measured as a disposition versus as an ability, and each kind of measurement elucidates an important facet of the construct. With regard to maximum-performance measures, it makes little difference whether one measures intercultural problem-solving in the participant’s own culture or a different one: The correlations between the two kinds of measures are very high. Cultural intelligence as an ability is related at some level to general intelligence but appears not to be the same thing. It draws on abstract reasoning, but also on tacit knowledge of strategies that are more or less effective in dealing with people from a different culture.

Our study was the third in a series (Sternberg et al. 2021, 2022), and the results across studies generally replicate each other. However, like all such studies, ours was not without weaknesses. First, the population of students tested was above average in academic skills and showed a lower standard deviation than would be the case for a broad sample of college students across the United States. In addition, that population of college students across the country would be narrower and less diverse, in any case, than a national sample of individuals. Second, the tests we used, although intended to sample a broad array of situations, of course could not sample the full range of high-stakes situations one might confront, such as what to do if an illegal immigrant knocks on one’s door. Third, cultural intelligence as displayed in high-stakes, emotionally fraught situations, such as confronting an illegal immigrant at one’s door, might be displayed in a manner different from that encountered when participants take tests online in the relative placidity of their home or dormitory. Fourth, some of the difficulties may be ones of problem-solving, in general, rather than of cultural problem-solving, in particular. A future study presenting both the present problems and comparable ones without the cultural component could address this conundrum. Fifth, the correlational pattern for both maximum- and typical-performance tests may reflect, in part, the well-established finding that, as in the emotional intelligence literature (Rivers et al. 2020) and the wisdom literature (Kunzmann 2019), maximum-performance tests tend to correlate with each other, and typical-performance measures tend to correlate with each other. Fifth, the predictive validity over time of our approach has yet to be shown. The typical-performance approach has shown some predictive validity (Schlaegel et al. 2021), but our approach still requires long-term predictive validation. At least some situational-judgment tests have been shown to be valid in measuring cultural intelligence (Chen 2020; Rockstuhl et al. 2015; Rockstuhl and Lievens 2021; see also Thomas and Inkson 2017). Finally, we cannot determine, at this point, the extent to which the kinds of skills measured by our tests might be teachable as opposed to only slightly modifiable or unmodifiable.

Nevertheless, we believe our study has a lot to offer in terms of showing that cultural intelligence appears to yield very similar rank orderings of people, whether the items are about how one would act in another culture or in one’s own culture with people of a different culture. In addition, based on our studies or other kinds of practical intelligence—intelligence as used in the world—we would expect some teachability as cultural intelligence draws heavily on tacit knowledge, which we learn from our interactions with the world (Sternberg and Hedlund 2002).

A lack of cultural intelligence is not benign. Cultural interaction today has become a sine qua non for people in positions of power. One has only to read or listen to media reports to learn how many allegedly educated persons in positions of power encourage xenophobia and negative affect toward foreigners. Some politicians base their appeal to their political base on disrespect or even disdain for foreigners, and some, such as Xi Jinping in China, Vladimir Putin in Russia, and Viktor Orban in Hungary, do not easily leave power once they attain it. If there ever has been a time when cultural intelligence and the ability to separate truth from falsehood about people of other cultures has been important, this would seem to be the time. We need to understand people from diverse cultures, and not be afraid of their differences. As a result, understanding, assessing, and teaching cultural intelligence may be more important now than ever before.

## Figures and Tables

**Table 1:** Descriptive statistics.

	N	Minimum	Maximum	Mean	Std. Deviation
Age	147	18	31	20.15	1.611
ACT	65	17	36	32.26	3.438
SAT Reading	94	500	800	721.76	59.770
SAT Math	94	500	800	766.01	49.680
SAT to ACT conversion	126	18	36	33.18	2.815
GPA	129	3	4	3.65	.373
Letter Sets	148	1	15	10.01	3.416
Figure Classification	148	3	106	66.03	20.934
CIB-Original	148	10.75	58.50	27.8074	8.19704
CIB-Modified	148	10.50	59.25	27.7213	8.52166
Total CI	148	23.00	117.75	55.5287	16.03908
VC Item1	148	.50	3.00	1.7247	.58666
VC Item2	148	.75	3.00	1.8851	.57022
VC Item3	148	.75	3.00	1.8809	.61985
CQS	148	34.00	140.00	90.3176	18.86978
CQS Dimension 1 MC	148	8.00	28.00	20.6216	4.07649
CQS Dimension 2 COG	148	6.00	42.00	22.3716	7.37183
CQS Dimension 3 MOT	148	7.00	35.00	24.4730	5.65979
CQS Dimension 4 BEH	148	5.00	35.00	22.8514	6.68572
HCT	135	22.00	60.00	41.6000	7.07233
TOPI	148	1.00	12.00	9.6081	2.75690
Cross Cultural Experience in Years	128	0	23	7.15	7.595
Number of Different Country Visited	147	0	36	7.15	6.795
Valid N (listwise)	28				

*Note*. Total CI = Sternberg Cultural Intelligence test with its subscales CIB-Original (“foreign culture”) and CIB-Modified (“own culture”); VC = Views on Culture; CQS = Cultural Intelligence Scale; CQS Dimension 1 MC = Metacognitive; CQS Dimension 2 COG = Cognitive; CQS Dimension 3 MOT = Motivational; CQS Dimension 4 BEH = Behavioral; HCT = Diagnosing Your Cultural Intelligence Test; TOPI = Test of Personal Intelligence.

**Table 2:** Internal consistency reliabilities.

Test	Coefficient Alpha Reliability	N of Items
Total CI	.975	24
CIB-Original	.957	12
CIB-Modified	.961	12
CQS	.929	20
HCT	.858	12
TOPI	.819	12
Letter Sets	815	15
Figure Classification	.960	112

*Note.* Total CI = Sternberg Cultural Intelligence test with its subscales CIB-Original (“foreign culture”) and CIB-Modified (“own culture”); CQS = Cultural Intelligence Scale; HCT = Diagnosing Your Cultural Intelligence Test; TOPI = Test of Personal Intelligence.

**Table 3:** Intercorrelations.

	1	2	3	4	5	6	7	8	9	10	11	12	13	14	15	16	17	18	19	20	21	22
ACT	1																					
SAT Reading	.54 **	1																				
SAT Math	.77 **	.32 **	1																			
SAT to ACT conversion	.95 **	.72 **	.71 **	1																		
GPA	.24	.39 **	.22*	.34 **	1																	
Letter Sets	.29 *	.06	.18	.20 *	.13	1																
Figure Classification	.38 **	.01	.17	.28 **	.04	.50 **	1															
CIB-Original	.09	.23 *	.15	.13	.14	.34 **	.22 **	1														
CIB-Modified	.09	.20	.09	.15	.06	.36 **	.23 **	.84 **	1													
Total CI	.09	.23 *	.13	.15	.10	.36 **	.24 **	.96 **	.96 **	1												
VC_Item 1	.16	.09	−.01	.09	.11	.41 **	.31 **	.47 **	.54 **	.52 **	1											
VC_Item 2	.06	.08	−.01	.03	.09	.23 **	.14	.35 **	.51 **	.45 **	.48 **	1										
VC_Item 3	.09	.21 *	.01	.12	.08	.25 **	.18 *	.44 **	.60 **	.54 **	.42 **	.49 **	1									
CQS	.23	−.02	.11	.10	−.01	−.01	.12	−.02	−.05	−.04	.03	.14	.10	1								
CQS Dimension 1 MC	.20	.10	.10	.13	.11	.02	.13	.07	.04	.06	.03	.16	.13	.77 **	1							
CQS Dimension 2 COG	.26 *	−.01	.13	.13	−.03	−.07	.01	−.16	−.19 *	−.18 *	−.07	.01	.02	.81 **	.51 **	1						
CQS Dimension 3 MOT	.09	−.03	−.02	.02	−.02	−.02	.04	.00	−.02	−.01	.06	.11	.05	.75 **	.50 **	.44 **	1					
CQS Dimension 4 BEH	.17	−.07	.11	.05	−.04	.07	.22 **	.09	.05	.07	.10	.19 *	.13	.82 **	.57 **	.51 **	.49 **	1				
HCT	.23	−.02	.09	.14	.00	.17 *	.26 **	−.05	−.01	−.03	.11	.15	.13	.79 **	.63 **	.50 **	.72 **	.71 **	1			
TOPI	.25 *	.08	.03	.18 *	.01	.55 **	.44 **	.38 **	.47 **	.45 **	.39 **	.41 **	.36 **	.04	.11	−.07	.06	.08	.10	1		
Cross Cultural Experience in Years	−.01	−.17	.03	−.07	−.27 **	−.07	.11	−.06	.04	−.01	.02	.04	−.01	.25 **	.18 *	.17	.15	.32 **	.18	.12	1	
Number of Different Countries Visited	.00	.11	.11	−.05	−.02	.04	.12	−.02	.00	−.01	.05	.17 *	.07	.26 **	.22 **	.12	.27 **	.22 **	.25 **	.20 *	.07	1

*Note.* *. Correlation is significant at the 0.05 level (two-tailed). **. Correlation is significant at the 0.01 level (two-tailed). Total CI = Sternberg Cultural Intelligence test with its subscales CIB-Original (“foreign culture”) and CIB-Modified (“own culture”); VC = Views on Culture; CQS = Cultural Intelligence Scale; CQS Dimension1 MC = Metacognitive; CQS Dimension2 COG = Cognitive; CQS Dimension3 MOT = Motivational; CQS Dimension4 BEH = Behavioral; HCT = Diagnosing Your Cultural Intelligence Test; TOPI = Test of Personal Intelligence.

**Table 4:** Rotated component matrix ^a^.

	Component		
	1	2	3
Letter Sets	.210	.824	−.003
Figure Classification	.042	.818	.176
Total CI	.744	.278	−.142
VC_Item1	.658	.384	.020
VC_Item2	.839	.047	.141
VC_Item3	.811	.084	.084
CQS	.045	−.015	.941
HCT	.041	.164	.930
TOPI	.446	.639	−.007
Extraction Method: principal component analysis. Rotation Method: varimax with Kaiser normalization. ^a^			

^a^. Rotation converged in five iterations. *Note*: Total CI = Sternberg Cultural Intelligence test; VC = Views on Culture; CQS = Cultural Intelligence Scale; HCT = Diagnosing Your Cultural Intelligence Test; TOPI = Test of Personal Intelligence.

**Table 5:** Rotated component matrix ^a^.

	Component		
	1	2	3
CIB-Original	.733	−.158	.197
CIB-Modified	.871	−.113	.093
VC_Item1	.706	.114	.007
VC_Item2	.745	.165	−.061
VC_Item3	.762	.100	.000
CQS	.033	.929	−.002
HCT	.059	.938	.028
TOPI	.591	.020	.116
SAT to ACT conversion	.084	.169	.816
GPA	.067	−.133	.797
Extraction Method: principal component analysis. Rotation Method: varimax with Kaiser normalization.^a^			

^a^. Rotation converged in four iterations. *Note:* CIB-Original = foreign culture; CIB-Modified = own culture; VC = Views on Culture; CQS = Cultural Intelligence Scale; HCT = Diagnosing Your Cultural Intelligence Test; TOPI = Test of Personal Intelligence.

**Table 6:** Rotated component matrix ^a^.

	Component		
	1	2	3
Total CI	.742	−.109	.139
VC_Item1	.742	.085	.018
VC_Item2	.798	.122	−.038
VC_Item3	.788	.071	.020
CQS	.030	.940	−.011
HCT	.070	.940	.026
TOPI	.621	−.009	.133
SAT to ACT conversion	.079	.166	.823
GPA	.072	−.142	.798
Extraction Method: principal component analysis. Rotation Method: varimax with Kaiser normalization.^a^			

^a^. Rotation converged in five iterations. *Note:* Total CI = Sternberg Cultural Intelligence test; VC = Views on Culture; CQS = Cultural Intelligence Scale; HCT = Diagnosing Your Cultural Intelligence Test; TOPI = Test of Personal Intelligence.

**Table 7:** Rotated component matrix ^a^.

	Component			
	1	2	3	4
CIB-Original	.747	.103	−.145	.226
CIB-Modified	.861	.170	−.108	.102
VC_Item1	.637	.334	.090	−.018
VC_Item2	.742	.122	.163	−.068
VC_Item3	.773	.089	.114	.010
CQS	.057	−.030	.935	.002
HCT	.030	.161	.927	.014
TOPI	.379	.678	−.064	−.015
SAT to ACT conversion	.002	.263	.154	.762
GPA	.108	−.049	−.114	.831
Letter Sets	.272	.743	−.008	.154
Figure Classification	.020	.831	.202	.088
Extraction Method: principal component analysis. Rotation Method: varimax with Kaiser normalization. ^a^				

^a^. Rotation converged in five iterations. *Note:* CIB-Original = foreign culture; CIB-Modified = own culture; VC = Views on Culture; CQS = Cultural Intelligence Scale; HCT = Diagnosing Your Cultural Intelligence Test; TOPI = Test of Personal Intelligence.

## Data Availability

Data are available upon request from the senior author.

## References

[B1-jintelligence-11-00212] Ang Soon, Ng Kok Yee, Rockstuhl Thomas, Sternberg Robert J. (2020). Cultural intelligence. Cambridge Handbook of Intelligence.

[B2-jintelligence-11-00212] Ang Soon, Van Dyne Linn, Koh Christine (2006). Personality correlates of the four-factor model of cultural intelligence. Group and Organization Management.

[B3-jintelligence-11-00212] Ang Soon, Van Dyne Linn, Rockstuhl Thomas, Gelfand Michele J., Chiu Chi-Yue, Hong Ying-Yi (2015). Cultural intelligence: Origins, conceptualization, evolution, and methodological diversity. Handbook of Advances in Culture and Psychology.

[B4-jintelligence-11-00212] Ang Soon, Van Dyne Linn, Koh Christine, Ng K. Yee, Templer Klaus J., Tay Cheryl, Chandrasekar N. Anand (2007). Cultural intelligence: Its measurement and effects on cultural judgment and decision making, cultural adaptation, and task performance. Management and Organization Review.

[B5-jintelligence-11-00212] Bennett Milton J. (1986). A developmental approach to training for intercultural sensitivity. International Journal of Intercultural Relations.

[B6-jintelligence-11-00212] Bennett Milton J., Kim Young Yun (2017). Developmental model of intercultural sensitivity. International Encyclopedia of Intercultural Communication.

[B7-jintelligence-11-00212] Budiman Abby (2020). Key Findings about U.S. Immigrants.

[B8-jintelligence-11-00212] Carroll John B. (1993). Human Cognitive Abilities: A Survey of Factor-Analytic Studies.

[B9-jintelligence-11-00212] Chen Xiaowen (2020). Different Paths, Same Destination? Comparison between Two Approaches to Developing Situational Judgment Tests on Cross Cultural Competency.

[B10-jintelligence-11-00212] Corbitt Nathan J. (1998). Global Awareness Profile (GAPtest).

[B11-jintelligence-11-00212] Deary Ian J. (2020). Intelligence: A Very Short Introduction.

[B12-jintelligence-11-00212] Earley P. Christopher, Mosakowski Elaine (2004). Cultural intelligence. Harvard Business Review.

[B13-jintelligence-11-00212] Ekstrom Ruth B., French J. W., Harman Harry Horace, Dermen D. (1976). Manual for Kit of Factor-Referenced Cognitive Tests.

[B14-jintelligence-11-00212] Hammer Mitchell R., Berg Michael Vande, Paige R. Michael, Lou Kris H. (2012). The Intercultural Development Inventory. Student Learning Abroad.

[B15-jintelligence-11-00212] Hammer Michael R., Bennett Milton J. (1998). The Intercultural Development Inventory (IDI) Manual.

[B16-jintelligence-11-00212] Hammer Mitchell R., Bennett Milton J., Wiseman Richard (2003). Measuring intercultural competence: The Intercultural Development Inventory. International Journal of Intercultural Relations.

[B17-jintelligence-11-00212] Harvard Program on Negotiation (2023). The Right Negotiation Environment: Your Place or Mine?. https://www.pon.harvard.edu/daily/negotiation-skills-daily/your-place-or-mine/.

[B18-jintelligence-11-00212] Kunzmann Ute, Sternberg Robert J., Glück Judith (2019). Performance-based measures of wisdom: State of the art and future directions. Cambridge Handbook of Wisdom.

[B19-jintelligence-11-00212] Mayer John D., Panter Abigail T., Caruso David R. (2018). Test of personal intelligence MINI MARKER-12 (TOPI MINI-12): Brief manual.

[B20-jintelligence-11-00212] McGrew Kevin S. (2009). CHC theory and the human cognitive abilities project: Standing on the shoulders of the giants of psychometric intelligence research. Intelligence.

[B21-jintelligence-11-00212] Rivers Susan E., Handley-Miner Isaac J., Mayer John D., Caruso David R., Sternberg Robert J. (2020). Emotional intelligence. Cambridge Handbook of Intelligence.

[B22-jintelligence-11-00212] Rockstuhl Thomas, Lievens Filip (2021). Prompt-specificity in scenario-based assessments: Associations with personality versus knowledge and effects on predictive validity. Journal of Applied Psychology.

[B23-jintelligence-11-00212] Rockstuhl Thomas, Ang Soon, Ng Kok-Yee, Lievens Filip, Van Dyne Linn (2015). Putting judging situations into situational-judgment tests: Evidence from intercultural multimedia SJTs. Journal of Applied Psychology.

[B24-jintelligence-11-00212] Schlaegel Christopher, Richter Nicole Franziska, Taras Vasyl (2021). Cultural intelligence and work-related outcomes: A meta-analytic examination of joint effects and incremental predictive validity. Journal of World Business.

[B25-jintelligence-11-00212] Schwarzenthal Miriam, Juang Linda P., Schachner Maja K., van de Vijver Fons J. R. (2019). A multimodal measure of cultural intelligence for adolescents growing up in culturally diverse societies. International Journal of Intercultural Relations.

[B26-jintelligence-11-00212] Stemler Steven, Imada Toshie, Sorkin Carolyn (2014). Development and validation of the Wesleyan Intercultural Competence Scale (WICS): A tool for measuring the impact of study abroad experiences. Frontiers: The Interdisciplinary Journal of Study Abroad.

[B27-jintelligence-11-00212] Sternberg Robert J. (2019). A theory of adaptive intelligence and its relation to general intelligence. Journal of Intelligence.

[B28-jintelligence-11-00212] Sternberg Robert J., Hedlund Jennifer (2002). Practical intelligence, g, and work psychology. Human Performance.

[B29-jintelligence-11-00212] Sternberg Robert J., Wong Chak Haang, Kreisel Anastasia P. (2021). Understanding and assessing cultural intelligence: Maximum-performance and typical-performance approaches. Journal of Intelligence.

[B30-jintelligence-11-00212] Sternberg Robert J., Siriner Ilaria, Oh Jaime, Wong Chak Haang (2022). Cultural intelligence: What is it and how can it effectively be measured?. Journal of Intelligence.

[B31-jintelligence-11-00212] Thomas David C., Inkson Kerr (2017). Cultural Intelligence: Surviving and Thriving in the Global Village.

[B32-jintelligence-11-00212] Van Dyne Linn, Ang Soon, Koh Christine, Ang Soon, Van Dyne Linn (2008). Development and validation of the CQS. Handbook of Cultural Intelligence.

[B33-jintelligence-11-00212] Van Dyne Linn, Ang Soon, Ngl Kok Yee, Rockstuhl Thomas, Tan Mei Ling, Koh Christine (2012). Sub-dimensions of the four factor model of cultural intelligence: Expanding the conceptualization and measurement of cultural intelligence. Social and Personality Psychology Compass.

